# Genetic differentiation in the southern population of the Fathead Minnow *Pimephales promelas* Rafinesque (Actinopterygii: Cyprinidae)

**DOI:** 10.7717/peerj.6224

**Published:** 2019-04-29

**Authors:** Nayarit E. Ballesteros-Nova, Rodolfo Pérez-Rodríguez, Rosa G. Beltrán-López, Omar Domínguez-Domínguez

**Affiliations:** 1 Programa Institucional de Doctorado en Ciencias Biológicas, Facultad de Biología, Universidad Michoacana de San Nicolás de Hidalgo, Morelia, Michoacán, México; 2 Laboratorio de Biología Acuática, Facultad de Biología, Universidad Michoacana de San Nicolás de Hidalgo, Morelia, Michoacán, México; 3 Laboratorio Nacional de Análisis y Síntesis Ecológica para la Conservación de Recursos Genéticos de México, Escuela Nacional de Estudios Superiores, Unidad Morelia, Universidad Nacional Autónoma de México, Morelia, Michoacán, México; 4 Laboratorio de Ictiología, Centro de Investigaciones Biológicas, Universidad Autónoma del Estado de Morelos, Cuernavaca, Morelos, México

**Keywords:** Mesa del Norte, Mexico, Genetic lineages

## Abstract

The North American cyprinid *Pimephales promelas* is a species with a wide distribution range, occurring in distinct hydrographic basins in Mexico, Canada, and the United States. Previous morphological and meristic analyses of *P. promelas* concluded that at least three subspecies exist in the midwestern and northeast region of the United States. No studies have been carried out on the Mexican population of *P. promelas*, but the findings of cryptic diversity in United States populations of this species, as well as in other codistributed fish species in Mexico could be an indication that Mexican populations of *P. promelas* consist of cryptic species. Using the mitochondrial gene cyt *b* and the first intron of the *S7* ribosomal protein-coding nuclear gene we carried out phylogenetic and phylogeographic analyses of populations of *P. promelas* across its distribution range in northwestern Mexico. Using this information were analyzed the structure and differentiation level between populations of *P. promelas* from distinct river basins in the region in identifying cryptic diversity. Twenty-four sequences were obtained for cyt *b*, and 30 for *S7,* which included the two heterozygous alleles. The results revealed the existence of four well-differentiated lineages: (1) Yaqui in the Pacific slope; (2) Santa Maria, and (3) Casas Grandes in the Guzman Basin; and (4) Nazas+Conchos in Chihuahua state. This challenges the current taxonomy of *P. promelas*. Differences in the relationships between markers and the small sample size for the Santa Maria population (*n* = 1), indicate that our results must be corroborated with more data and morphological analyses. Biogeographic analysis of these findings suggest that the evolutionary history of *P. promelas* is associated with the fragmentation of the ancestral Rio Grande river system since Miocene in northwestern Mexico consistent with findings for codistributed fish species.

## Introduction

Phylogeographic studies provide useful information that complements biogeographic hypotheses relating to the current distributions of fish species and their link to fragmentation and/or to expansion events (Devitt, 2006; Riddle & Hafner, 2006; Vallinoto et al., 2010; Schönhuth et al., 2015).

In North America, tectonic activity and climate changes are the main factors that have contributed to the formation of drainage basins and the evolution of associated species (Galloway, Whiteaker & Ganey-Curry, 2011; Aranda-Gómez et al., 2018). In northwestern Mexico, the main tecto-volcanic activity since the Oligocene is related to the formation of the Sierra Madre Occidental (SMO) mountain range (Ferrari, Valencia-Moreno & Bryan, 2007; Aguirre-Díaz et al., 2008). This high level of tectonic activity associated with the SMO has been proposed as responsible for the high level of endemism of flora and fauna in this region of Mexico, and the main force that has shaped the speciation process (vicariance and/or dispersion) in freshwater fishes of the region (Echelle et al., 2005; Hughes, Rinne & Calamusso, 2005; Ceballos, Arroyo-Cabrales & Ponce, 2010; Morrone, 2010; Schönhuth & Mayden, 2010; Domínguez-Domínguez et al., 2011; Schönhuth et al., 2011; Schönhuth et al., 2012; Schönhuth et al., 2014; Schönhuth et al., 2015; Clements, Bart & Hurley, 2012). The SMO is also considered an important biogeographic corridor and a Pleistocene refuge (Hughes, Rinne & Calamusso, 2005), associated with the expansion and contraction of numerous species ranges in response to climate change during the Pleistocene (Domínguez-Domínguez et al., 2011).

**Figure 1:**
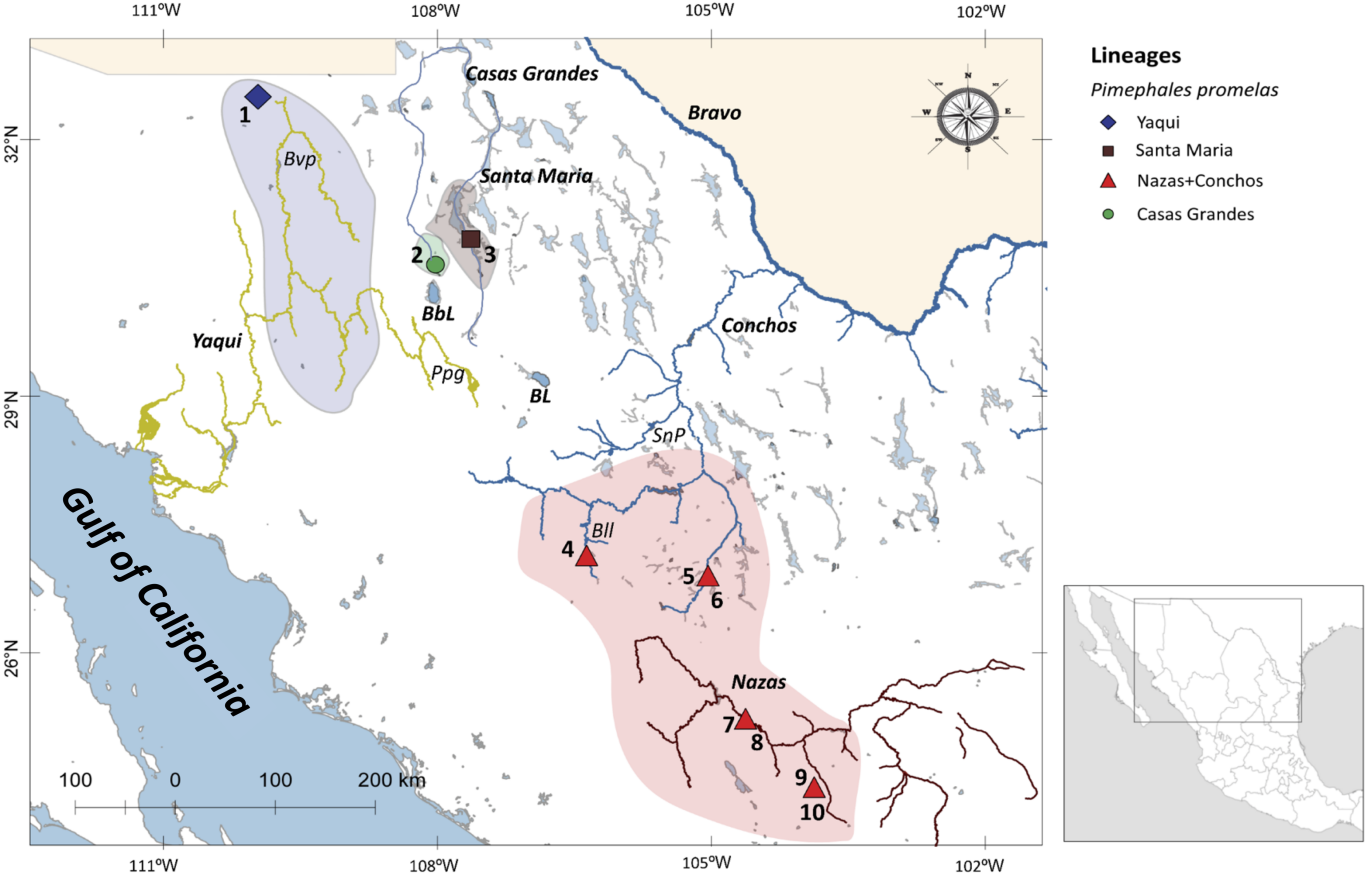
Drainage basins sampled for *P. promelas* and genetic lineages found. Colors and shapes correspond to the four lineages identified in phylogenetic analyses. Numbers and forms indicate different localities where *P. promelas* was collected: 1. Cabullona, (six samples); 2. Casas Grandes, (five samples); 3. Buenaventura, (one sample); 4. Nonoava, (one sample); 5. Villa Coronado, (two samples); 6. Porvenir, (two samples); 7. Jicorica, (two samples); 8. Abasolo, (two samples); 9. Paso Nacional, (two samples); and 10. Covadonga, (two samples), according to the localities in Table S1. Abbreviations: *Bvp*, Bavispe River; *BbL*, Babicora Lagoon; *Ppg*, Papigochic River; *BL*, Bustillos Lagoon; *SnP*, San Pedro River; *Bll*, Balleza River.

Northwestern Mexico is an area including two major physiographic provinces: the highland Chihuahuan desert region in the Mesa del Norte, and the SMO (Schönhuth et al., 2015). The Mesa del Norte is located from the United States (US)-Mexico border to Zacatecas and San Luis Potosi states (Miller, Minkley & Norris, 2005). These areas include major drainages as Yaqui, Mayo, Fuerte, and Mezquital rivers on the Pacific Slope of the SMO; endorheic drainages as the Nazas and Aguanaval rivers; and the Atlantic drainage Conchos River (Schönhuth et al., 2015) (Fig. 1).

Previous studies of fishes in the Mesa del Norte based on molecular data (Smith & Miller, 1986; Mayden, Matson & Hillis, 1992; Mayden et al., 1992; Echelle et al., 2005; Domínguez-Domínguez et al., 2011; Schönhuth et al., 2011; Schönhuth et al., 2012; Schönhuth et al., 2015; Corona-Santiago et al., 2018) and geological information (Galloway, Whiteaker & Ganey-Curry, 2011), proposed the existence of an extended Rio Grande system that originated in the Oligocene and persisted for more than 10 million years. Extensive zones of the southwestern highlands of the US (Colorado Plateau, in current Utah, Colorado and Arizona), Chihuahuan Desert (New Mexico, Chihuahua, Coahuila and Durango) and the current Lower Grande River Basin were part of the ancient Rio Grande system (Galloway, Whiteaker & Ganey-Curry, 2011; Schönhuth et al., 2015).This paleo-river system was proposed to have connected drainages in Mexico that are currently independent (Mayden, Matson & Hillis, 1992; Echelle et al., 2005; Schönhuth, Doadrio & Mayden, 2007; Schönhuth et al., 2011; Schönhuth et al., 2015; Domínguez-Domínguez et al., 2011; Corona-Santiago et al., 2018), acting as a hydrological corridor for the ancestors of current freshwater fish species and permitting their expansion across the drainages in the Chihuahuan Desert (Smith & Miller, 1986; Mayden, Matson & Hillis, 1992; Domínguez-Domínguez et al., 2011; Schönhuth et al., 2011; Schönhuth et al., 2015). This scenario includes the possible transfers and/or integration of headwaters of the Mesa del Norte with drainages from the Pacific slope (Yaqui, Mayo, Fuerte, Piaxtla, Culiacan and Mezquital rivers) (Minckley, Hendrickson & Bond, 1986; Miller, Minkley & Norris, 2005; Domínguez-Domínguez et al., 2011; Schönhuth et al., 2011; Schönhuth et al., 2014; Schönhuth et al., 2015; Corona-Santiago et al., 2018) as far south as the Nazas and Aguanaval rivers (Smith & Miller, 1986; Mayden, Matson & Hillis, 1992; Domínguez-Domínguez et al., 2011; Schönhuth et al., 2011; Schönhuth et al., 2014; Schönhuth et al., 2015; Corona-Santiago et al., 2018).

One of the most widespread fish species in the Mesa del Norte hydrological basins is the Fathead Minnow *Pimephales promelas*. This species is distributed from eastern North America into Northern Mexico. *P. promelas* ranges from Lake Slave to the Hudson Bay at its northern limit, southward through the Mississippi Valley, the Great Plains and the Gulf slope streams of Alabama and the Grande River basin into the Conchos River. The range of *P. promelas* also includes the endorheic basins of Casas Grandes, Nazas, Del Carmen, Santa Maria, and Bustillos in the Mesa del Norte, as well as the Pacific slope drainage of the Yaqui River in Mexico (Vandermeer, 1966; Miller, Minkley & Norris, 2005). Previous morphological analyses of *P. promelas* populations in the United States concluded that at least three subspecies of this widespread species exist in the midwestern and northeast region of the US (Vandermeer, 1966). No studies have been carried out in Mexican population, but based on the findings of cryptic diversity in United States populations of this species (Vandermeer, 1966), as well as in other codistributed fish species in Mesa del Norte (*Campostoma anomallum* (Blum et al., 2008), *Cyprinella lutrensis* (Schönhuth & Mayden, 2010), *Campostoma ornatum* (Domínguez-Domínguez et al., 2011; Schönhuth et al., 2011), *Dionda episcopa* (Schönhuth et al., 2012), *Codoma ornata* (Schönhuth et al., 2015) and *Pantosteus plebeius* (Corona-Santiago et al., 2018) we hypothesize that Mexican populations of *P. promelas* have been taxonomically underestimated.

In accordance with the above, the aim of the present study is to assess the genetic divergences in *P. promelas* populations, to tests the current taxonomic status with molecular data, and determine whether populations from distinct basins correspond to different evolutionary lineages. Based on mitochondrial and nuclear markers, we use both, phylogenetic and phylogeographic approaches to examine the populations of *P. promelas* across its distribution range in northwestern Mexico.

## Methods

### Taxon sampling

The specimens of *P. promelas* were collected from five independent drainage basins and ten localities across the Mexican distributional range of the species using electrofishing and seine netting techniques: (1) Cabullona, (6 specimens); (2) Casas Grandes, (5 specimens); (3) Buenaventura, (1 specimen); (4) Nonoava, (1 specimen); (5) Villa Coronado, (2 specimens); (6) Porvenir, (2 specimens); (7) Jicorica, (2 specimens); (8) Abasolo, (2 specimens); (9) Paso Nacional, (2 specimens); and (10) Covadonga, (2 specimens) (Fig. 1; Table S1). For each specimen we fixed a piece of the fin in 95% ethanol for extraction of the DNA, which was then stored at −70 °C. We preserved a maximum of five specimens per site in 5% formalin following the protocols approved by the Mexican Ministry of Environment and Natural Resources (SEMARNAT). We deposited fish and tissue samples in the fish collection of the Universidad Michoacana de San Nicolas de Hidalgo, Mexico (SEMARNAT registration number MICH-PEC-227-07-09). All procedures were reviewed and approved by a committee of Mexican Ministry of Environment and Natural Resources, under collection permit number PPF/DGOPA-362/1. *C. ornata*, *Pimephales notatus*, and *Pimephales tenellus* were used as outgroups (based on prior phylogenetic studies (Schönhuth et al., 2008; Schönhuth et al., 2016; Schönhuth et al., 2018) (Table S1).

### DNA extraction, PCR amplification, and sequencing

DNA was extracted using the standard proteinase K/phenol/chloroform protocol (Sambrook, Fritsch & Maniatis, 1989). We obtained sequences for a fragment of the mitochondrial cytochrome *b* gene (cyt *b*: 1,049 bp) in twenty-four specimens using the primers LA (5′-GTGACTTGAAAAACCACCGTTG) and HA (3′-CAACGATCTCCGGTTTACAAGAC) (Dowling et al., 2002). A subset of eighteen specimens was selected, accounting for all the variation found for the cyt *b* gene, for amplification of the first intron of the nuclear *S7* ribosomal protein-coding gene (*S7*: 704 bp), with the primers S71F (5′-TGGCCTCTTCCTTGGCCGTC) and S72R (3′- AACTCGTCTGGCTTTTCGCC) (Chow & Hazama, 1998).

The final concentrations in each 25 µL polymerase chain reaction (PCR) were: 50 ng template DNA, 10 µM of each primer, 0.7 units of Taq DNA polymerase, 0.25 mM of each dNTP, 2.5 µL of Reaction Buffer and 2.5 mM MgCl_2_. Thermocycling conditions for amplification of the mitochondrial cyt *b* gene consisted of an initial denaturalization step of 3 min at 94 °C: followed by 35 cycles of 30 s at 94 °C, 1 min at 48 °C, 90 s at 72 °C, and a final 5 min extension step at 72 °C. The *S7* gene was amplified under the following conditions: initial denaturalization step of 3 min at 94 °C, followed by 35 cycles of 45 s at 94 °C, 50 s at 57 °C, 100 s at 72 °C, and a final step of 10 min extension at 72 °C. All PCR products were purified with ExoSAP-IT™. The purified PCR products were sent to Macrogen Korea for sequencing.

The sequences were edited and aligned using the default parameters of Clustal X (Thompson et al., 1997) implemented in Mega v6.06 (Tamura et al., 2013) and examined using chromatograms. The *S7* sequences were phased with point mutation using DNAsp v5.10 (Librado & Rozas, 2009). We evaluated nuclear recombination using the pairwise homoplasy index (PHI) test (Bruen, Philippe & Bryant, 2006) as implemented in Splitstree4 (Huson & Bryant, 2006). No significant recombination was detected in the nuclear *S7* sequences (*p* = 0.3741). The sequences of *S7* showed heterozygous indels; in this case we performed a manual reconstruction of the two allele phases following the procedure described by Sousa-Santos et al. (2005).

### Phylogenetic analyses and haplotype networks

We obtained the evolutionary substitution models, based on the corrected Akaike Information Criterion (AICc), and an optimal partition setting using PartitionFinder v1.1.0 (Lanfear et al., 2012). We obtained the optimal partition setting by assigning a substitution model to each gene. The models obtained were the Transitional Model (TIM3) (Posada, 2003) + gamma (TIM3+G) for the cyt *b* gene and the Tamura-Nei model (Tamura & Nei, 1993) + gamma (TrN+G) for the *S7* gene.

Bayesian Inference (BI) analyses were applied in MrBayes v3.2.1 (Ronquist et al., 2012). The sequences were analyzed in two different data sets, one for each gene independently and one for both genes concatenated. We used the two heterozygous alleles for the *S7* gene. The analyses for 10 million generations were run with two independent runs, implementing four Markov Chain Monte Carlo (MCMC) processes and sampling every 500 generations. We evaluated the convergence of the chains with the log-likelihood (-InL) values of the two independent runs on Tracer v1.5 (Rambaut & Drummond, 2007), discarding 10% of the generations as burn-in to construct the consensus tree. We visualized the trees in FigTree v1.4.2 (Rambaut, 2014).

Maximum Likelihood (ML) trees were constructed in RAxMLGUI v1.3.1 (Silvestro & Michalak, 2012; Stamatakis, 2014), as implemented in CIPRES (Miller, Pfeiffer & Schwartz, 2010), with the default GTR+G+I model using the rapid bootstrap algorithm with 1000 replicates (CIPRES portal v3.3) at the San Diego Supercomputer Center at http://www.phylo.org/sub_sections/portal/.

For each gene, we constructed unrooted networks under a null hypothesis of no genetic differentiation among populations, using the median-joining method (Leigh & Bryant, 2015) as implemented in PopART v1.7 (available at http://popart.otago.ac.nz).

### Species tree analysis, genetic distances and divergence times

We estimated a species tree and divergence times for the major nodes in *Pimephales promelas* for both genes (cyt *b* + *S7*) using the Bayesian Method in *BEAST v1.8.1 (Drummond et al., 2012). The substitution models were set according to the model selected for each gene by PartitionFinder v1.1.0 (Lanfear et al., 2012), the Generalised Time Reversible model (Tavare, 1986) + gamma (GTR+G) for the cyt *b* gene and the TN93 model (Tamura & Nei, 1993) + gamma (TN93+G) for the individual *S7* gene. We carried out this analysis with a subset of 33 sequences that include all different haplotypes for all genes, and two outgroup sequences (*P. notatus* and *P. tenellus* based on a previous phylogenetic study (Schönhuth et al., 2018)) (Table S1). The lineages in the *BEAST analysis were selected according to the lineages recovered on the phylogenetic trees. The model parameters were unlinked across cyt *b* and *S7* genes. Considering that the performance of the strict clock is virtually identical to the lognormal distribution, of the uncorrelated relaxed clock (Firth et al., 2010), and the use of uncorrelated relaxed clocks takes the rate variation among lineages into account (Baele et al., 2012; Hasegawa, Kishino & Yano, 1985), we selected a lognormal relaxed clock (Uncorrelated) model for branch length (Drummond et al., 2006). Because of the lack of reliable fossil data, we calibrated the molecular clock using the mutation rate of cyt *b* in teleosts of 0.76–2.2%/million years (Zardoya & Doadrio, 1999; Berendzen, Gamble & Simons, 2008; Nagle & Simons, 2012), applied in a prior with a uniform distribution. Since the mutation rate is not available for the nuclear gene, we included this gene in the analysis without calibration information. We selected the tree prior-species Tree: Yule process model. We ran Markov chain Monte Carlo analysis for 120 million generations, sampled every 1000 generations. We ran analyses in CIPRES Science Gateway v3.3 (http://www.phylo.org/sub_sections/portal/). We assessed whether parameter values had reached effective sample size and convergence in Tracer v1.5 (Rambaut & Drummond, 2007), and built the maximum clade credibility tree using Tree Annotator v1.8.1 (Drummond et al., 2012), discarding the first 10% of the trees as burn-in. We visualized the tree in FigTree v1.4.2 (Rambaut, 2014).

Previous phylogenetic studies in freshwater fishes in northwestern Mexico use uncorrected pairwise (*p*)-distances for generating distance matrices (Domínguez-Domínguez et al., 2011; Schönhuth et al., 2011; Schönhuth et al., 2014; Schönhuth et al., 2015; Corona-Santiago et al., 2018). In order to be able to compare our results we used *p-* distances, calculated among the recovered monophyletic groups in the phylogenetic trees for both genes independently in Mega v6.06 (Tamura et al., 2013).

### Bayesian species delimitation test

We conducted Bayesian multilocus species delimitation tests using the concatenated dataset in Bayesian Phylogenetics and Phylogeography (BPP v3.1; Yang & Rannala, 2010; Yang & Rannala, 2014; Yang, 2015). This method uses a species phylogeny represented by a user-specified guide tree and accommodates lineage sorting due to ancestral polymorphism (Yang & Rannala, 2010). To generate the guide tree, each lineage recovered in the concatenated phylogenetic analyses (BI, ML, and species tree) was treated as a terminal taxon in *BEAST (Drummond et al., 2012), with the resulting species tree used as a guide for the BPP analyses.

For the BPP analyses of the two concatenated genes, we used the reversible-jump Markov Chain Monte Carlo (rjMCMC) (Yang & Rannala, 2010) algorithm to determine whether to collapse or retain nodes throughout the phylogeny. Using the entire dataset coded by each gene, we tested with the Analysis A10 algorithm, in which we used the rjMCMC algorithm to move between species delimitation models that were compatible with a fixed guide tree (Rannala & Yang, 2013; Yang & Rannala, 2010).

To determine whether lineages could be considered as distinct species under a general lineage species concept, the program assessed the probability of the node separating the species (De Queiroz, 2007). We used algorithm 0 with values of 5, 10 and 15 for the fine-tuning parameter in order to ensure that the rjMCMC mixed effectively in the species delimitation models. We conducted analyses with prior distributions on the ancestral population size (*θ*) and root age (*τ*_0_) (Leaché & Fujita, 2010) to discern how these parameters influenced the results. We initially set the gamma prior at *θ* and *τ*_0_ to the values *α* = 1 and 2, and *β* = 10, 100, and 2000; and ran five analyses of each with different starting seeds for two independent chains of 500,000 generations with a burn-in of 50,000 and thinning conducted every five generations.

## Results

We obtained 54 sequences for both genes: 24 for cyt *b*, and 30 for *S7*, which included the two heterozygous alleles.

We obtained 16 haplotypes from the 24 sequences of the cyt *b* gene. These haplotypes were defined by 213 polymorphic sites within a 1049 bp sequence fragment (total number of mutations = 240). Sixty-nine of those sites were singletons and 164 substitutions were parsimony informative.

We obtained 18 haplotypes for the *S7* gene. These haplotypes were defined by 43 polymorphic sites (total number of mutations = 45). Twenty-five of those sites were singletons and 18 substitutions were parsimony informative.

### Phylogenetic relationships and haplotype networks

**Figure 2:**
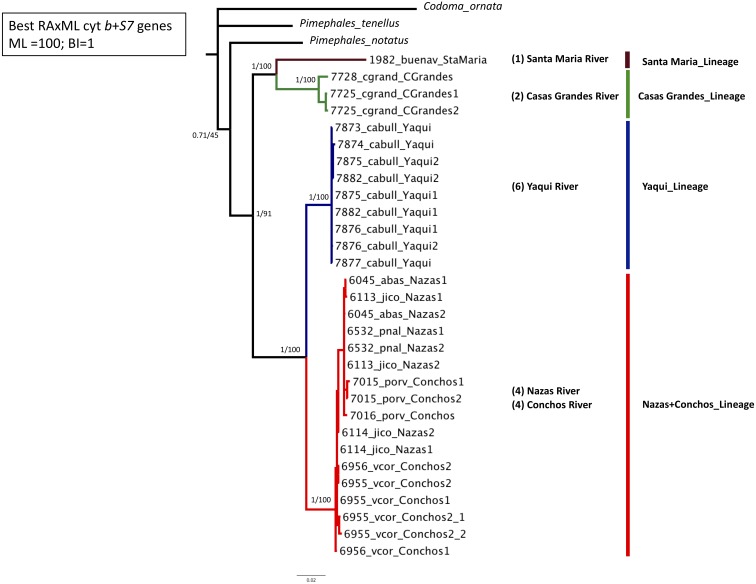
Maximum Likelihood (ML) phylogeny of *P. promelas* based on the concatenated genes (cyt *b* + *S7*), using a GTR+G+I model with ML bootstrap values (based on 1,000 replicates). Numbers on the branches separated by a diagonal correspond to Bayesian posterior probabilities and Maximum Likelihood bootstrap values. Numbers in parentheses correspond to the sample size in each drainage basin. Lineages are color-coded according to the distribution areas in the map (Fig. 1).

Phylogenetic analyses based on the two concatenated genes (cyt *b* +*S7*) (1,753 bp) recovered four well-supported lineages: Santa Maria lineage, clustered one specimen from the Santa Maria River in the Guzman Basin (BS = 100% and PP = 1); Casas Grandes lineage, clustered two specimens from the Casas Grandes River in the Guzman Basin (BS = 100% and PP = 1); Yaqui lineage clustered six specimens from the Yaqui River in the Cabullona locality (BS = 100% and PP = 1); and, Nazas+Conchos lineage, clustered eight specimens from the Nazas and Conchos rivers (BS = 100% and PP = 1) (Fig. 2; Table S1).

Phylogenies based on separate analyses for each gene revealed generally consistent results, although they recovered a differing number of lineages. Phylogenies based on cyt *b* and concatenated analyses recovered four major lineages and the same phylogenetic relationships: Santa Maria lineage (BS = 100% and PP = 1); Casas Grandes lineage (BS = 100% and PP = 1); Yaqui lineage (BS = 100% and PP = 1); and Nazas+Conchos lineage, included specimens from localities of the Lower Conchos Drainage (Villa Coronado, El Porvenir and Nonoava), as well as specimens from localities of the Nazas River drainage (Covadonga, Abasolo, Paso Nacional and Jicorica) (BS = 97% and PP = 1) (Fig. 1 and Fig. S1; Table S1). The phylogeny recovered with *S7* identified similar lineages: Casas Grandes, BS = 98% and PP = 1; Santa Maria, BS = 96% and PP = 1; Nazas+Conchos, BS = 97% and PP = 1; and Yaqui, BS = 48% and PP = 0.66. In the *S7* analysis the Santa Maria lineage was recovered as a sister group to a well-supported clade including all three lineages, Casas Grandes, Nazas+Conchos and Yaqui (Fig. S2).

**Figure 3:**
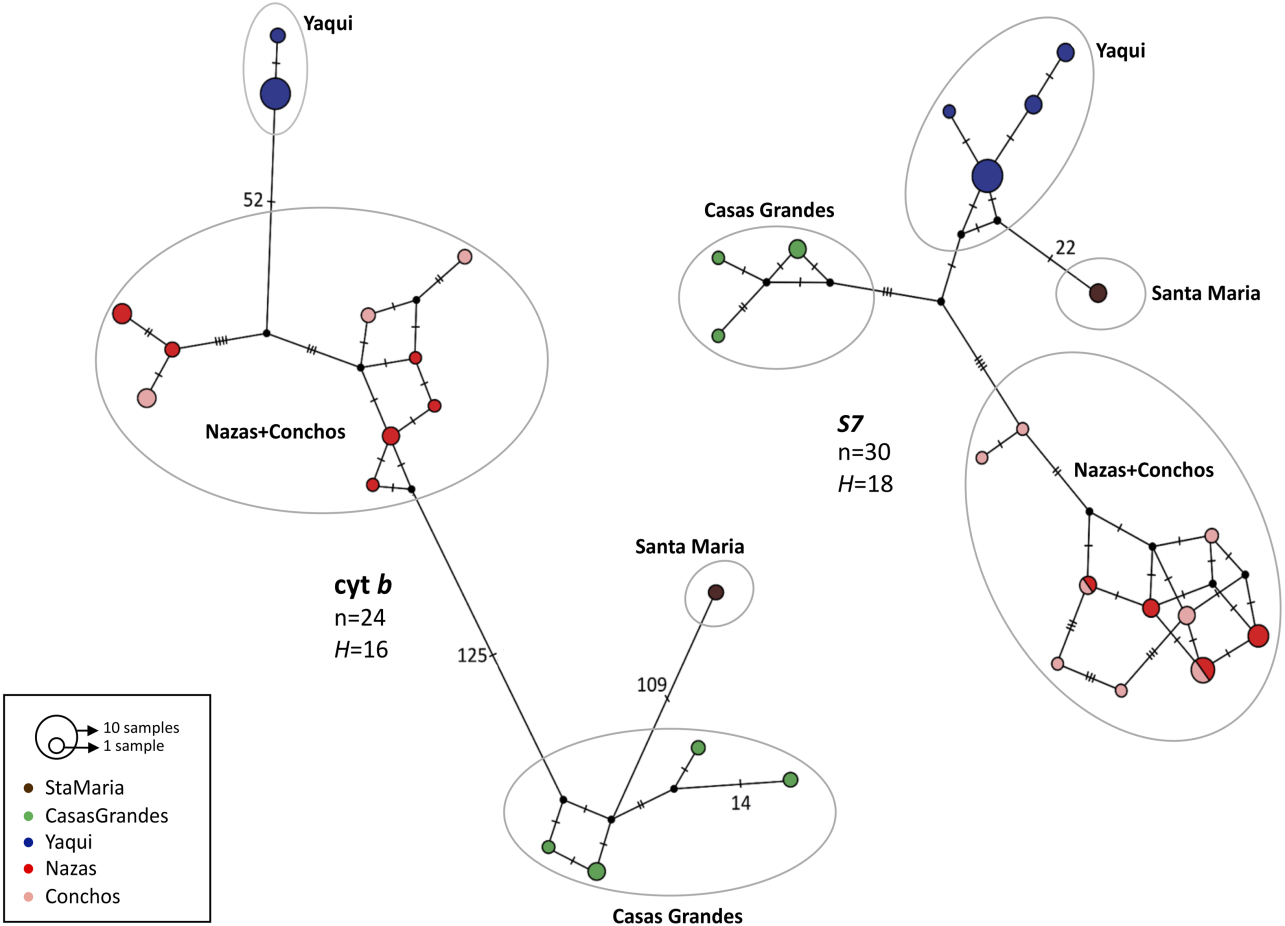
Median-joining haplotype network for mitochondrial (cyt *b*) and nuclear (intron *S7*) genes for *P. promelas*. Each circle represents a different haplotype; circle sizes are proportional to the number of individuals with a particular haplotype. Small black dots represent missing (unsampled or extinct) haplotypes. Lines between circles represent one mutational step, and numbers presented are the number of mutations between haplotypes. Haplogroups are color-coded according to the distribution areas in the map (Fig. 1).

For both genes, the haplotype networks showed the existence of four haplogroups, corresponding to the four lineages found in the phylogenetic analyses: Casas Grandes, Santa Maria, Nazas+Conchos and Yaqui lineages; however, as in the phylogenetic analyses, different relationships were found between the cyt *b* and *S7* groups (Fig. 3).

For cyt *b* we found 52 mutation steps (MS) between Yaqui and Nazas+Conchos lineages, 109 MS between Casas Grandes and Santa Maria lineages, and 125 MS between Nazas+Conchos and Casas Grandes lineages. For the Nazas+Conchos population we found nine haplotypes: three in Conchos Basin (Villa Coronado, Nonoava and El Porvenir localities), six in Nazas Basin, one in Covadonga and Paso Nacional localities and two in each of the Jicorica and Abasolo localities. For the Yaqui population we found two haplotypes in the Cabullona locality. In the Casas Grandes population we found four haplotypes. In the Santa Maria population we found one haplotype (Fig. 3).

For *S7* we found a mixture of haplotypes among the Nazas and Conchos samples. We found the Santa Maria lineage to be separated by 22 MS from the Yaqui lineage. For the Nazas+Conchos population we found ten haplotypes: six in Conchos Basin (two in the El Porvenir locality and four in the Villa Coronado locality), two in Nazas Basin (Abasolo and Jicorica localities). For the Yaqui population we found four haplotypes in the Cabullona locality. For the Casas Grandes population we found three haplotypes (Fig. 3).

### Species tree analysis, divergence times and genetic distances

**Figure 4:**
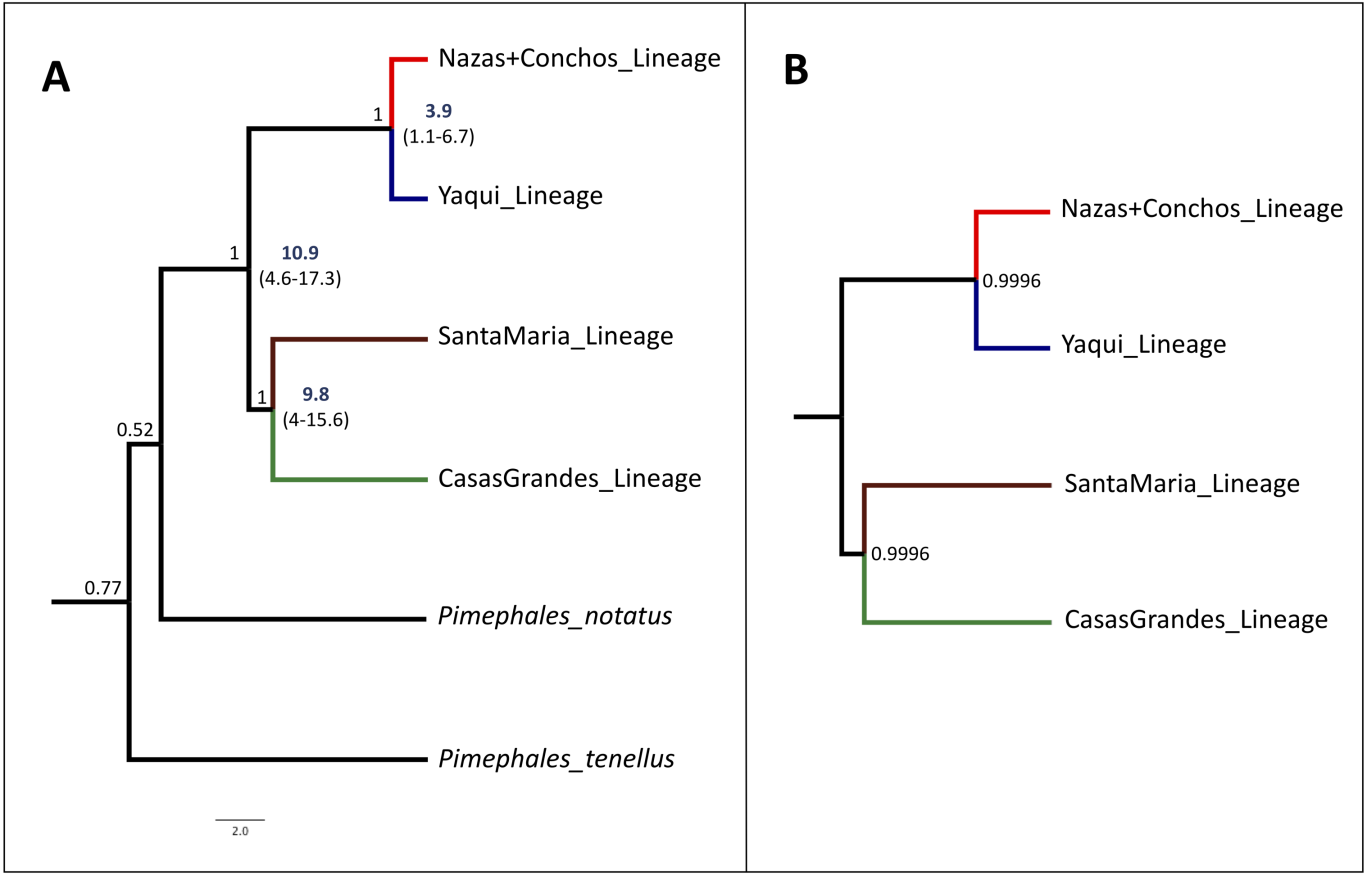
Time-Calibrated Species-Tree and Bayesian species delimitation test results for *P. promelas.* Both nuclear and mitochondrial gene sequences were used. Lineages are color-coded according to the distribution areas in the map (Fig. 1). *(A)* Time-calibrated Species-Tree phylogeny and Divergence Time estimates for nodes based on substitution rates for the cyt *b* gene of 0.76–2.2%/ million of years (Zardoya & Doadrio, 1999; Berendzen, Gamble & Simons, 2008; Nagle & Simons, 2012). Since the mutation rate is not available for the nuclear gene we included in the analysis without calibration information. Numbers in bold and values in parentheses represent the 95% highest posterior density of divergence time estimates. Values on the branches represent the posterior probability. *(B)* Bayesian species delimitation test, assuming four species according to the guide tree obtained from *BEAST. We considered speciation probability values >0.95 as strong support for a speciation event. For the five analyses, we applied different combinations of *θ* and *t*_0_ priors: 1. *θ* = (*α*:2, *β*:2000), *t*_0_ = (*α*:2, *β*:20000); 2. *θ* = (*α*:2, *β*:20), *t*_0_ = (*α*:2, *β*:200); 3. *θ* = (*α*:2, *β*:20), *t*_0_ = (*α*:2, *β*:20000); 4. *θ* = (*α*:2, *β*:200), *t*_0_ = (*α*:2, *β*:20000) and, 5. *θ* = (*α*:2, *β*:20), *t*_0_ = (*α*:2, *β*:200). We obtained strong support (posterior probability ≥0.99) in all the five analyses. The number in the nodes correspond to the major posterior probability value obtained in all the five analyses.

The species tree analysis with the concatenated dataset (cyt *b* +*S7)* supports the assumption of four lineages of high internal branch-length Bayesian Posterior Probability (>99%), as seen in the cyt *b* and the concatenated (cyt *b* +*S7*) phylogenetic trees. This result is also consistent with the *S7* gene phylogenetic analyses, corresponding to the four recovered lineages (Fig. 4A).

The divergence between the main clades of *P. promelas* was dated to the Mid-Miocene and Mid-Pliocene. The separation of the main clades (Nazas+Conchos and Yaqui *vs.* Santa Maria and Casas Grandes) was dated at *ca*. 10.9 Million years ago (Mya) (95% Highest Posterior Density (HPD): 4.6–17.3) (Fig. 4A). The split between the Santa Maria and Casas Grandes lineages was estimated at *ca*. 9.8 Mya (95% HPD: 4–15.6), whereas the separation event of the Yaqui and Nazas+Conchos lineages was estimated at *ca*. 3.9 Mya (95% HPD: 1.1–6.7) (Fig. 4A).

The maximum genetic distance for both genes occurred between the Nazas+Conchos and Santa Maria lineages (10.6% for cyt *b* and 4.7% for *S7*) (Tables 1A and 1B). The minimum genetic distance for the cyt *b* gene was found between the Nazas+Conchos and Yaqui lineages (3.7%). For *S7* the minimum genetic distance occurred between the Casas Grandes and Yaqui lineages (1.1%). Genetic distances within each lineage ranged from 0% to 0.5% with cyt *b* and 0.1 to 0.4 with *S7* (Tables 1A and 1B).

**Table 1:** Genetic divergences within *P. promelas*. Ranges of uncorrected pairwise sequence divergence (%) between major lineages within Pimephales promelas. Values in parentheses correspond to the sample size in each lineage. Values in bold correspond to the genetic distance within each lineage. (A) Genetic divergences in mitochondrial cyt *b* gene. (B) Genetic divergences in the first intron of the S7 ribosomal protein-coding gene.

(A)				
Lineages	Nazas+Conchos (13)	Yaqui (6)	Casas Grandes (5)	Santa Maria (1)
Nazas+Conchos	**0.4%**			
Yaqui	3.7%	**0%**		
Casas Grandes	9.4%	9.6%	**0.5%**	
Santa Maria	10.6%	10.5%	7.8%	–

(B)				
Lineages	Nazas+Conchos (8)	Yaqui (6)	Casas Grandes (2)	Santa Maria (1)
Nazas+Conchos	**0.4%**			
Yaqui	1.4%	**0.1%**		
Casas Grandes	1.7%	1.1%	**0.3%**	
Santa Maria	4.7%	3.6%	4.4%	–

### Bayesian species delimitation test

The speciation model based on the species tree estimate strongly supported the assumption of four putative species. We obtained strong support (posterior probability ≥ 0.99) for the tested speciation model of four *a priori* defined species within Mexican populations of *P. promelas* (Casas Grandes lineage, Santa Maria lineage, Yaqui lineage, and Nazas+Conchos lineage (Fig. 4B)). The BPP was not sensitive to species delimitation, and no alteration of posterior probabilities of the speciation model was observed when we applied different values of root age (*τ*_0_) and population size (*θ*), demonstrating high posterior probabilities for model tested with the A10 algorithm.

## Discussion

The results presented herein revealed that southern populations of *P. promelas* distributed across northwestern Mexico correspond to at least four well-supported independent evolutionary lineages. The four lineages show geographic congruence, with the occurrence of each divergent lineage in independent hydrographic basins, as have been found in other widely distributed fish species (Blum et al., 2008; Schönhuth & Mayden, 2010; Domínguez-Domínguez et al., 2011; Schönhuth et al., 2011; Schönhuth et al., 2012; Schönhuth et al., 2014; Schönhuth et al., 2015; Corona-Santiago et al., 2018). This study is consistent with other studies of the freshwater fish species distributed along the Mesa del Norte in Mexico that have found that many species previously considered as a single widespread taxon, are in fact a species complex (e.g., *C. ornatum* (Domínguez-Domínguez et al., 2011; Schönhuth et al., 2011), and *C. ornata* (Schönhuth et al., 2015)). This study is also consistent with other studies of *P. promelas*, such as those carried out on the US populations by Vandermeer (1966) with morphological characters, that show the species to be made up of complexes of cryptic species.

### Phylogenetic relationships and taxonomic implications

In this study of Mexican *P. promelas* populations the results of the concatenated phylogenetic trees (Fig. 2), species tree (Fig. 4A) and BPP analyses (Fig. 4B), supports a strong genetic differentiation pattern, that corresponds to four well-supported lineages, that must be considered independent evolutionary lineages and even undescribed species (Fig. 4). These four evolutionary lineages were recovered in both genes in spite of the incongruence in the phylogenetic relationships between markers (Figs. S1 and S2) and the small sample size in the case of the Santa Maria Basin (*n* = 1) population. The incongruence found between concatenated and independent genes genealogies could not be attributed to retention of ancestral polymorphisms due to incomplete lineage sorting and/or introgression following secondary contact, since we did not recover shared haplotypes between the four well-differentiated lineages in either of the two genes (Fig. 3, Figs. S1 and S2). But the low sample size in the Santa Maria Basin (*n* = 1) could generate gene trees bias results, (Satta, Klein & Takahata, 2000; Kopp & True, 2002; Rokas et al., 2003; Rokas & Carroll, 2005), accordingly we recommend a revaluation of the phylogenetic relationships with a larger sample size and more nuclear loci.

In the case of the genetic divergences, we also found congruences in the differentiation pattern of highly differentiated lineages. For cyt *b* among the four lineages the genetic divergences ranged between 3.7% among the Yaqui and Nazas+Conchos lineages, to 10.6% among populations of the Nazas+Conchos and Santa Maria lineages. These genetic distances are the same or greater than those found among the six species of the Chihuahuan Desert Group of the genus *Gila,* that range from 3.68 to 5.56 (Schönhuth et al., 2014) and minimum distances of 1.3% have been found between southwestern species in the *Dionda* genus (Schönhuth et al., 2008), 1.9% between species in *Cyprinella*, and 4.7% in species of the genus *Tampichthys* (Schönhuth & Mayden, 2010) and 2.1–4.0% between species of *Algansea* (Pérez-Rodríguez et al., 2009). Moreover, the *S7* gene showed relatively high genetic differences for a nuclear gene, with minimum genetic distances (*p*-distance 1.1%) found in the comparison between the geographically proximate Casas Grandes and Yaqui lineages, and maximum distances (*p*-distance 4.7%) in the Santa Maria and Nazas+Conchos lineages. A previous study has found similar genetic distances in the *S7* nuclear gene for a species in the Cyprinidae family and in species of the genus *Algansea*, in which interspecific genetic distances between 0.5 to 3.6% were found for *S7* (Pérez-Rodríguez et al., 2009).

Accordingly, the Mexican populations of *P. promelas* shows four independent evolutionary lineages that could be recognized as different species. A more integrative taxonomic analysis is pending in order to complement the results presented herein.

#### Biogeographic implications derived from the cladogenetic pattern

Widespread species are associated with suitable biological and ecological traits that permit a high dispersal ability and an ability to colonize new habitats (Nathan, 2001). In the case of freshwater fishes, the discontinuity of aquatic habitats represents the main barrier to range expansion (Pérez-Rodríguez et al., 2015). In the case of northwestern Mexico, tecto-volcanic events since the Miocene and Pleistocene, glacial/interglacial cycles, and increasing regional aridity since the Holocene, fragmented the ancestral Rio Grande system (Metcalfe, 2006; Schönhuth et al., 2015), effectively isolating different populations and shaping the geographic distribution and phylogenetic relationships of the freshwater fishes of the region (Smith & Miller, 1986; Wood & Mayden, 2002; Blum et al., 2008; Schönhuth & Mayden, 2010; Domínguez-Domínguez et al., 2011; Schönhuth et al., 2011; Schönhuth et al., 2012; Schönhuth et al., 2014; Schönhuth et al., 2015; Aranda-Gómez et al., 2018; Corona-Santiago et al., 2018).

**Figure 5:**
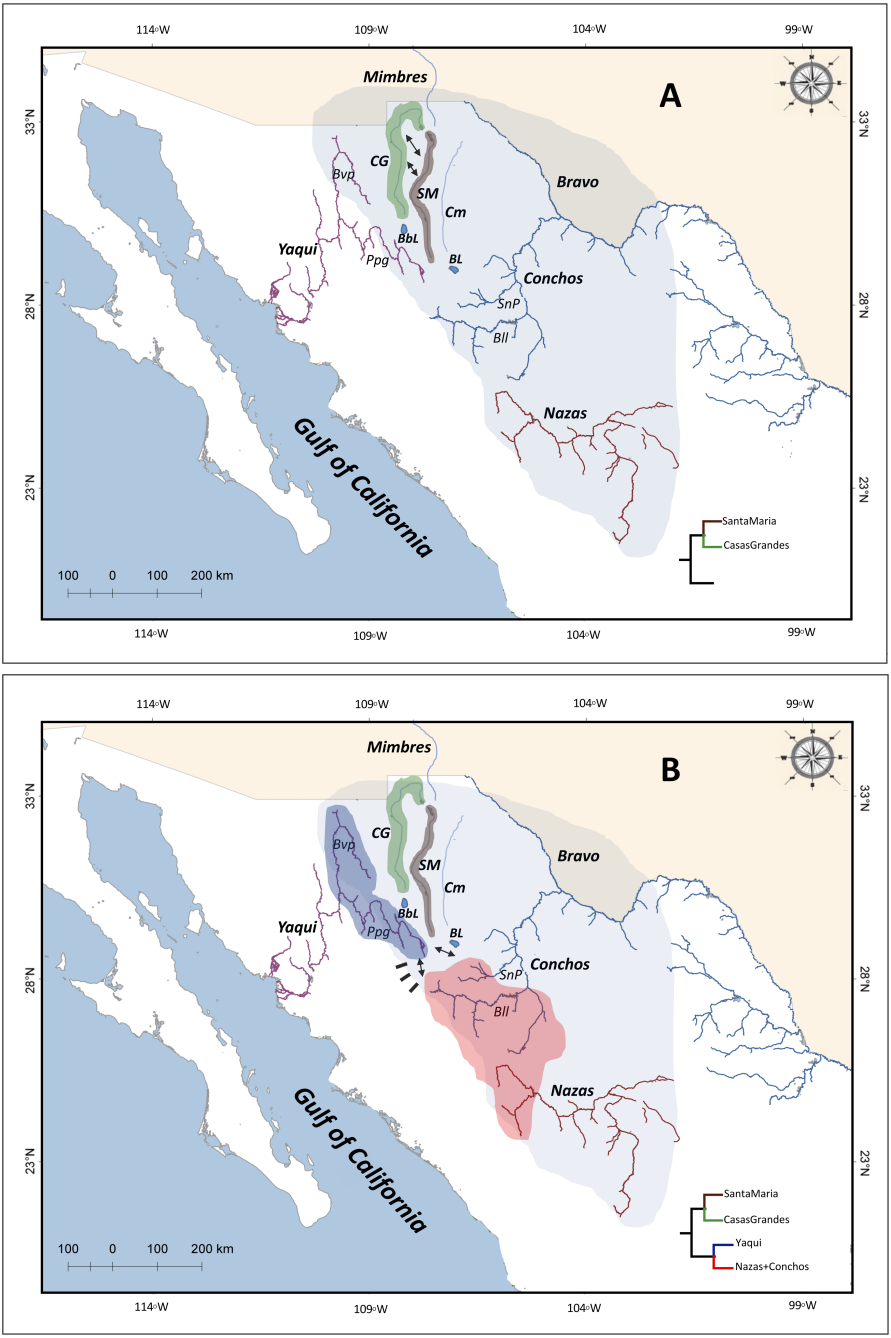
Chronosequence of cladogenetic events, and suggested drainage basin configurations and distributions of the common ancestor of the four *P. promelas* lineages. (A) Ancient ‘Rio Grande system’; separation of the Santa Maria Lineage, related to the regional patterns of uplift and subsidence during the Miocene and Pliocene. (B) Hydrological connectivity between areas across the SMO (Yaqui-Conchos rivers); separation between Yaqui *vs.*Nazas+Conchos lineages, related to the tecto-volcanic episodes in SMO evolution. ** Green, brown, blue and red shaded correspond to the distribution of the lineages as shown in the phylogenetic trees. Black rectangles represent tecto-volcanic events. Solid arrows represent river capture. The colored shade of light blue represents the hypothetical area of the ancient ’Rio Grande System’, as described by Schönhuth et al. (2011). Abbreviations: *CG*, Casas Grandes River; *SM*, ** Santa Maria River; *Cm*, ** Del Carmen River; *Bvp*, ** Bavispe River; *BbL*, ** Babicora Lagoon; *Ppg*, ** Papigochic River; *BL*, ** Bustillos Lagoon; *Bll*, ** Balleza River; *SnP*, ** San Pedro River.

Our divergences times are in agreement with the paleohydrological history of the region in space and time. Accordingly, in the present study, the most recent common ancestor of the four *P. promelas* lineages and the separation of the Santa Maria and Casas Grandes lineages were dated to the Mid-Miocene (*ca.* 10.9 Mya, 95% HPD: 4.6–17.3; *ca.* 9.8 Mya, 95% HPD: 4.0–15.6 respectively) (Fig. 4A). The most plausible biogeographic scenario for the isolation of these lineages is the fragmentation of the ancestral Rio Grande system (Smith, Song & Miller, 1984; Albritton, 1958; Miller & Smith, 1986; Minckley, Hendrickson & Bond, 1986; Smith & Miller, 1986; Schönhuth et al., 2015), caused by the regional patterns of uplift and subsidence during the Miocene and Pliocene, while an arid climate extended across the western interior, causing the reorganization of drainage configurations (Galloway, Whiteaker & Ganey-Curry, 2011; Schönhuth et al., 2015) (Fig. 5A). This pattern of isolation has also been proposed for codistributed species such as the genus *Tampichthys* (endemic to central-east Mexico) and its sister group *Codoma* (north-western distribution) occurring during the Mid-Miocene, around 9.17 Mya or 12.19 Mya (Schönhuth et al., 2015).

The separation between the Yaqui and the Nazas+Conchos lineages was dated to the Pliocene, approximately 3.9 Mya (95% HPD: 1.1–6.7) (Fig. 4A). This event seems to be related to the tecto- volcanic episodes in SMO evolution, including repeat alkaline basalt events (Henry & Aranda-Gómez, 2000; Aranda-Gómez et al., 2005; Ferrari, Valencia-Moreno & Bryan, 2007), river capture and peripheral isolation (Ferrari, Valencia-Moreno & Bryan, 2007; Aguirre-Díaz et al., 2008) (Fig. 5B), as has been hypothesized for those Pacific slope rivers that have headwaters extending eastward in the SMO to areas of the Mesa del Norte. Previous studies identified the possible transfers of fish species between drainage basins by integration or fragmentation of drainages, as is the case of the Yaqui River, and have documented closely related lineages on both sides of the SMO, in the Yaqui and Conchos rivers. Smith & Miller (1986) suggested that rivers in Northwestern Mexico, draining to the Pacific (Upper Yaqui and Upper Mezquital) across desert regions, originated through headwater capture from the ancestral and extant Rio Grande system, and caused fish dispersal by stream capture events (Schönhuth et al., 2011).

A mixture of samples from the current Nazas and Conchos drainage basins was found within the Nazas+Conchos lineage of *P. promelas*. This could be related to a recent connection of both basins via river capture or through pluvial lakes, as shown by the cyt *b* gene, whereas the mixture of haplotypes between both populations in *S7* could be related to incomplete lineage sorting due to the low mutation rate of nDNA. The close relationship between samples of both drainages was previously found in *C. ornatum* populations, a relationship explained by a fish interchange as a result of river capture (Schönhuth et al., 2011). Similarly, Burr (1976), in a previous review of *C. ornatum*, hypothesized the formation of a connection between the Nazas River and the Grande River via the Mayran and Viesca lagoons (both currently dry) during the Late Pleistocene (Meek, 1904; Burr, 1976). While a previous biogeographic study in *C. ornata* found a close relationship between the Conchos and Nazas drainage populations, which was attributed to incomplete lineage sorting, no biogeographic scenario was presented (Schönhuth et al., 2015).

## Conclusions

The geographic distribution of the four genetic lineages recovered in *P. promelas* (Casas Grandes, Santa Maria, Yaqui, and Nazas+Conchos) is similar to the lineage distributions found in other freshwater fishes in the North of Mexico, such as *C. ornatum* (Domínguez-Domínguez et al., 2011; Schönhuth et al., 2011), *Rhinichthys cataractae* (Kim & Conway, 2014), *C. ornata* (Schönhuth et al., 2015), and *P. plebeius* (Corona-Santiago et al., 2018). Cladogenic events in *P. promelas* are hypothesized to have been caused by the combined influence of tectonic events and increasing regional aridity; in particular, the fragmentation of the ancestral Rio Grande system and interchange events between basins via stream capture.

The phylogenetic analyses, species tree and Bayesian species delimitation tests results validate the presence of four genetic lineages. In the future, it would be interesting to increase both the sample size and the number of genes, and to evaluate the morphological diversity in the other Mexican populations of *P. promelas*. This would allow the taxonomic status of the genetic lineages found in the present study to be established and the determination of their relationship with other populations of *P. promelas* in North America.

##  Supplemental Information

10.7717/peerj.6224/supp-1 Figure S1 Maximum Likelihood (ML) Phylogeny of *Pimephales promelas* based on the cyt *b* gene, using a GTR+G+I model with ML bootstrap values (based on 1000 replicates) Numbers on the branches separated by a diagonal correspond to Bayesian posterior probabilities and Maximum Likelihood bootstrap values. Numbers in parentheses correspond to the sample size in each drainage basin. Lineages are color-coded according to the distribution areas in the map (Fig. 1).

10.7717/peerj.6224/supp-2 Figure S2 Maximum Likelihood (ML) Phylogeny of *Pimephales promelas* based on the first intron of the *S7* ribosomal protein-coding gene, using a GTR+G+I model with ML bootstrap values (based on 1000 replicates) Numbers on the branches separated by a diagonal correspond to Bayesian posterior probabilities and Maximum Likelihood bootstrap values. Numbers in parentheses correspond to the sample size in each drainage basin. Lineages are color-coded according to the distribution areas in the map (Fig. 1). ****

10.7717/peerj.6224/supp-3 Table S1 Sampling localities for the specimens of *Pimephales promelas* and outgroups analyzed in this study Collection numbers are listed for vouchers stored at institutional collections followed by tissue numbers. Dr: Drainage. Mex: Mexico. SLUM: Saint Louis University, St. Louis, Missouri, USA. CPUM, Universidad Michoacana de San Nicolás de Hidalgo, Michoacán, Mexico. MNCN, Museo Nacional de Ciencias Naturales, Madrid, Spain.

10.7717/peerj.6224/supp-4 Supplemental Information 1 Raw data - cyt b gene alignment

10.7717/peerj.6224/supp-5 Supplemental Information 2 Raw data - S7 alignment

## Additional Information and Declarations

### Field Study Permissions

The following information was supplied relating to field study approvals (i.e., approving body and any reference numbers): All procedures were reviewed and approved by a committee of Mexican Ministry of Environment and Natural Resources (SEMARNAT), under collection permit number PPF/DGOPA-362/1.

### Data Availability

The following information was supplied regarding data availability: The raw data are provided in the Supplemental Files and in GenBank: MK821253–MK821306.
