# Supplementary figures and images for: Genetic differentiation in the southern population of the Fathead Minnow Pimephales promelas Rafinesque (Actinopterygii: Cyprinidae)
Source: PeerJ. 2019 Apr 29;7:e6224. doi: 10.7717/peerj.6224 (PMC6497047; doi:10.7717/peerj.6224)

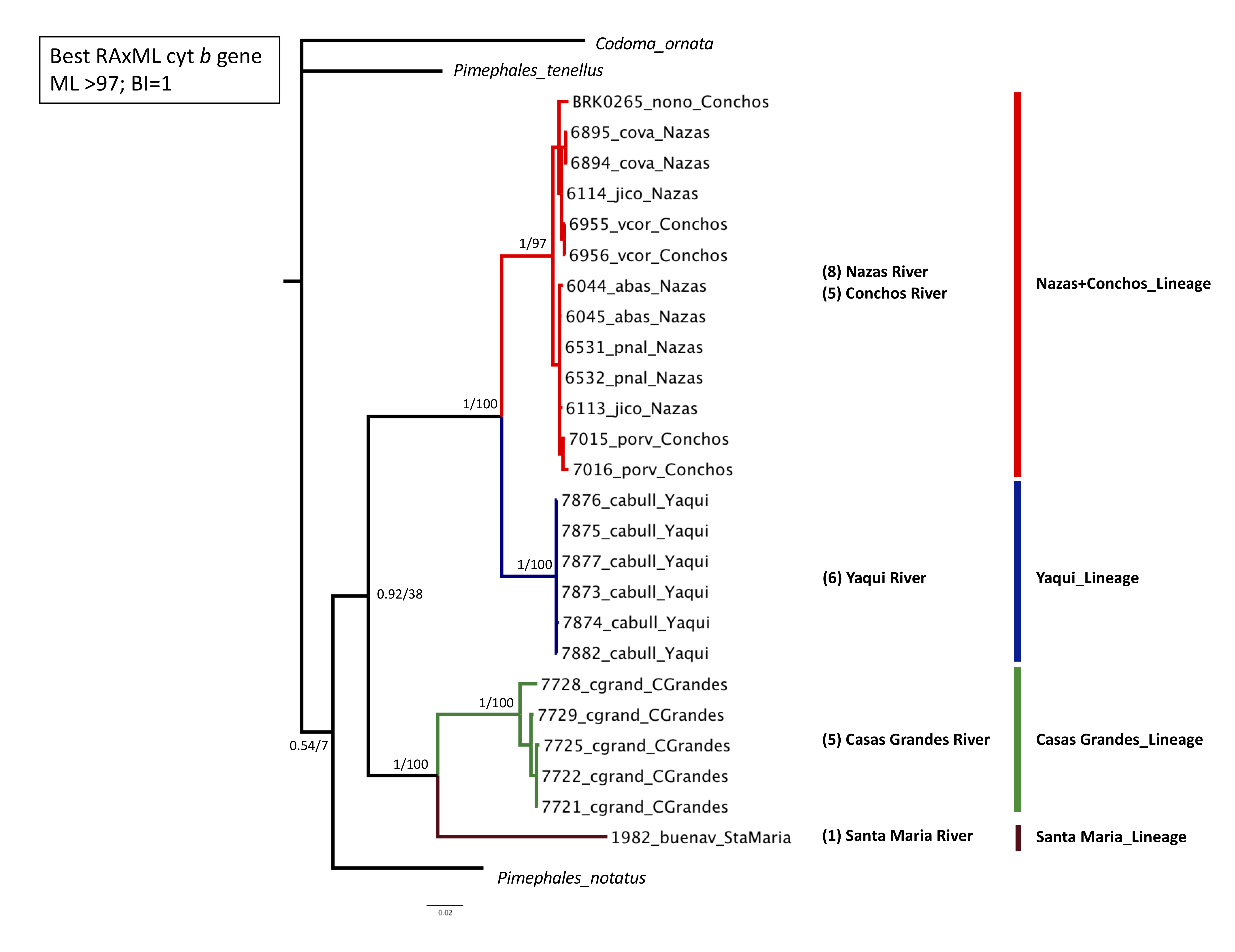

Supplement: Figure S1 — Numbers on the branches separated by a diagonal correspond to Bayesian posterior probabilities and Maximum Likelihood bootstrap values. Numbers in parentheses correspond to the sample size in each drainage basin. Lineages are color-coded according to the distribution areas in the map (Fig. 1). [file peerj-07-6224-s001.png]

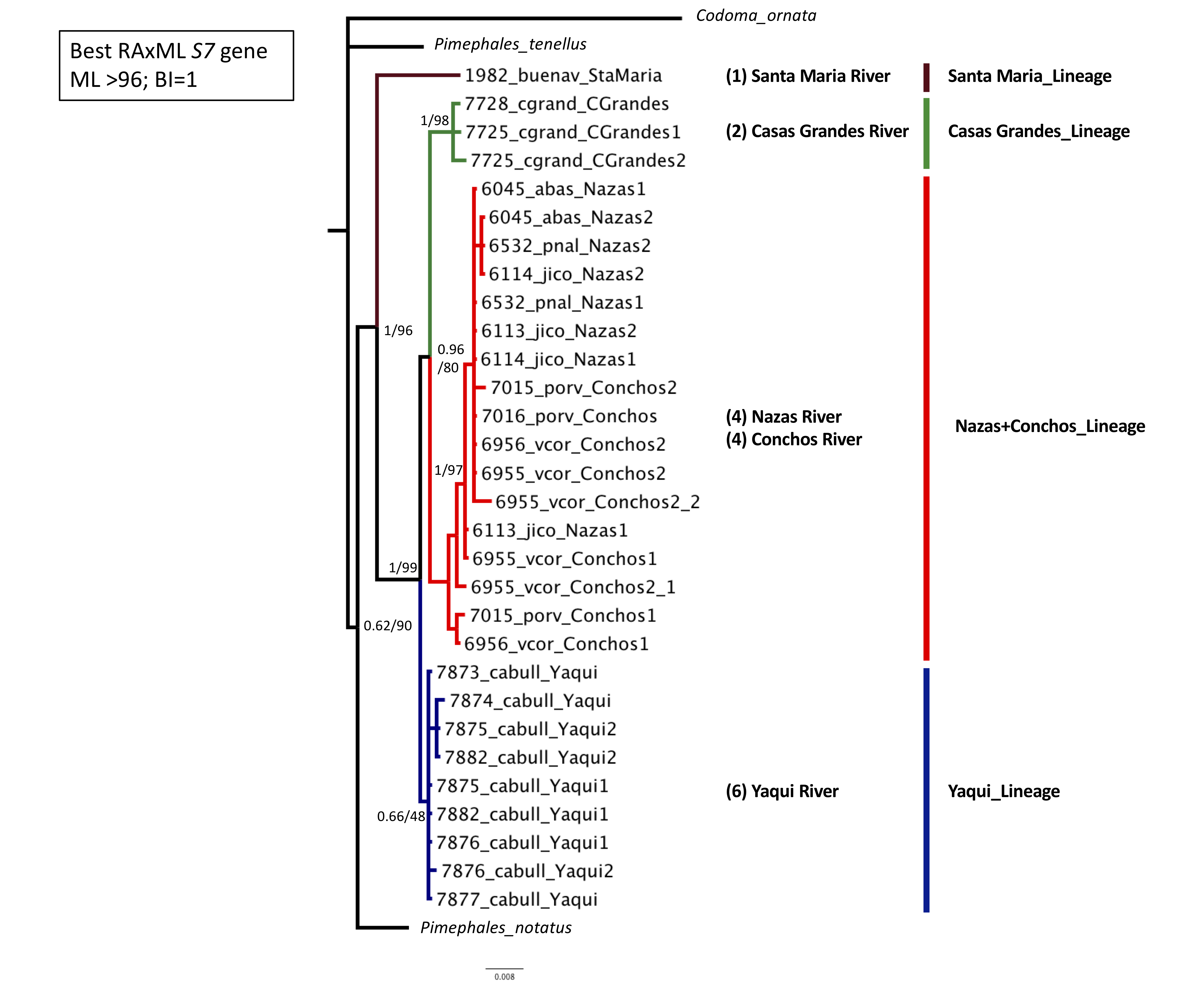

Supplement: Figure S2 — Numbers on the branches separated by a diagonal correspond to Bayesian posterior probabilities and Maximum Likelihood bootstrap values. Numbers in parentheses correspond to the sample size in each drainage basin. Lineages are color-coded according to the distribution areas in the map (Fig. 1). [file peerj-07-6224-s002.png]
