# Supplementary material for: Genetic differentiation in the southern population of the Fathead Minnow Pimephales promelas Rafinesque (Actinopterygii: Cyprinidae)
Source: PeerJ. 2019 Apr 29;7:e6224. doi: 10.7717/peerj.6224 (PMC6497047; doi:10.7717/peerj.6224)
Supplement: Table S1 — Collection numbers are listed for vouchers stored at institutional collections followed by tissue numbers. Dr: Drainage. Mex: Mexico. SLUM: Saint Louis University, St. Louis, Missouri, USA. CPUM, Universidad Michoacana de San Nicolás de Hidalgo, Michoacán, Mexico. MNCN, Museo Nacional de Ciencias Naturales, Madrid, Spain. [file peerj-07-6224-s003.docx]

|  | **LOCALITY** | **BASIN** | **Cyt *b*** | ***S7*** | ***GPS coordinates*** |
| --- | --- | --- | --- | --- | --- |
| ***Pimephales promelas*** | Cabullona River, road Agua Prieta City-Nacozari de García City, Sonora, Mex. | Yaqui | CPUM7882, CPUM7873-CPUM7877  (6) | CPUM7882, CPUM7873-CPUM7877  (6) | 31º7′59.5”N,  109º33′51.3″W |
|  | Nazas River, at El Peñasco, San Rafael Jicorica Town, Durango, Mex. | Nazas | CPUM6113-CPUM6114  (2) | CPUM6113-CPUM6114  (2) | 25º22′59.7″N,  104º45′54″W |
|  | Abasolo | Nazas | CPUM6044-CPUM6045  (2) | CPUM6045  (1) | -- |
|  | Covadonga River, at Peñón Blanco Town, Durango, Mex. | Nazas | CPUM6894-CPUM6895  (2) | -- | 24º43′55.8″N,  104º5′23.2″W |
|  | Paso Nacional | Nazas | CPUM6531-CPUM6532  (2) | CPUM6532  (1) | 25º16′31.6″N,  104º0′57.7″W |
|  | El Porvenir River, at Peñón Blanco Town, Durango, Mex. | Conchos | CPUM7015-CPUM7016  (2) | CPUM7015-CPUM7016  (2) | 26º55′23.6″N,  106º19′46.3″W |
|  | Nonoava River, Chihuahua, Mex. | Conchos | BRK02-65  (1) | -- | -- |
|  | Florido River, at Villa Coronado Town, Chihuahua, Mex. | Conchos | CPUM6955-CPUM6956  (2) | CPUM6955-CPUM6956  (2) | 26º44′2.2″N,  105º8′5.2″W |
|  | Casas Grandes River at Hacienda San Diego, S of Casas Grandes City, Chihuahua, Mex. | Casas Grandes | CPUM7728-CPUM7729, CPUM7721-CPUM7722, CPUM7725  (5) | CPUM7725, CPUM7728  (2) | 30º14′22.2″N,  107º50′57.5″W |
|  | Buenaventura | Santa María | MNCN1982  (1) | MNCN1982  (1) | -- |
| **Outgroups** |  |  |  |  |  |
| ***Pimephales tenellus*** | CottonWood River at Emporia, Lyon Co., Neoso River, Kansas, USA. | Kansas | SLUM5542.01 | SLUM5542.01 | -- |
| ***Pimephales notatus*** | Little Vermilion R @ Hwy 63 (upstream) just NW Newport R., Mississippi, Ohio Basin. | Ohio | PN3508 | PN3508 | -- |
| ***Codoma ornata*** | Isolated pool Arroyo de los Alcoces, Conchos Dr., Chihuahua, Mex. | Conchos | BRK02-64 | BRK02-64 | -- |
